# Supplementary material for: A method for semantic textual similarity on long texts
Source: PeerJ Comput Sci. 2025 Sep 19;11:e3202. doi: 10.7717/peerj-cs.3202 (PMC12453783; doi:10.7717/peerj-cs.3202)
Supplement: Supplemental Information 1 — Supporting information for noise analysis, criteria for evaluation of datasets, criteria for generation of datasets’ gold standard, and configuration of parameters used with the language models. Results of configurations tested with language models. [file peerj-cs-11-3202-s001.pdf]

Supplemental information and data for a method for semantic textual similarity on long texts

1 Analysis of distributions

The purpose of testing the identical documents separately is to empirically measure the noise level in the more similar documents to mitigate false positives and negatives up to the performance of the employed model. The distributions of the comparisons of each document with itself are shown in Figure 1. The largest number of pairs of sentences are grouped from the first decile to the sixth for all the document pairs due to the low degree of similarity. The distributions for the systematic *2,627* dataset comparisons from *72* documents in Figure 2 also show high concentrations in the lower deciles. Therefore, we can empirically set the noise (2) of the document pairs ν = *0.4*[1]. The soundness of each document pair (5) is shown in Figure 3, the number of sentence pairs with a level of similarity equal or above are less than 1×10^3, while the space of sentence pairs can reach 1×10^5 in the greatest case, which represents the *1%* of the combinations of sentence pairs. This analysis provides empirical evidence that attention is not required to be fully implemented; instead, selective attention reduces time and resources for establishing the similarities between the two texts.

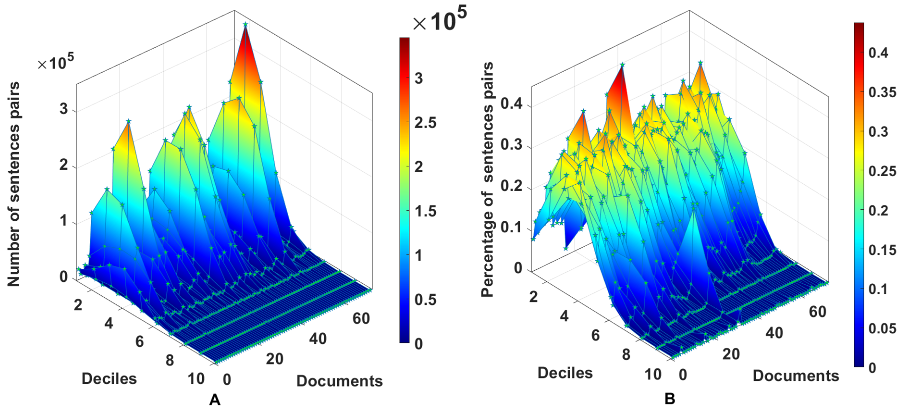

Figure 1: Distribution by deciles of comparisons of documents against themselves, using the model all-MiniLM-L6-v2. All document pairs are considered identical.

[1]The estimation of ν varies with the distribution of similarity produced by each model; the distribution depends on the criteria for training the models, having in mind that non-related pairs of texts produce non-zero degree of similarity from the embeddings. The initial estimation may be inaccurate. The proposed method aids to set a value that minimizes the bias of ν to improve the estimation of similarity.

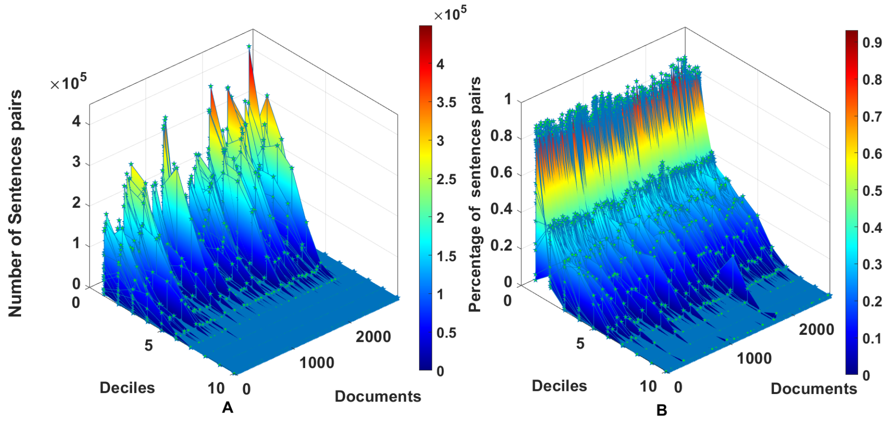

Figure 2: Distribution of pairs of documents within the dataset of *72* documents, using the model all-MiniLM-L6-v2. The number of text pairs is *2,627*, including the *72 self-comparisons* for each document.

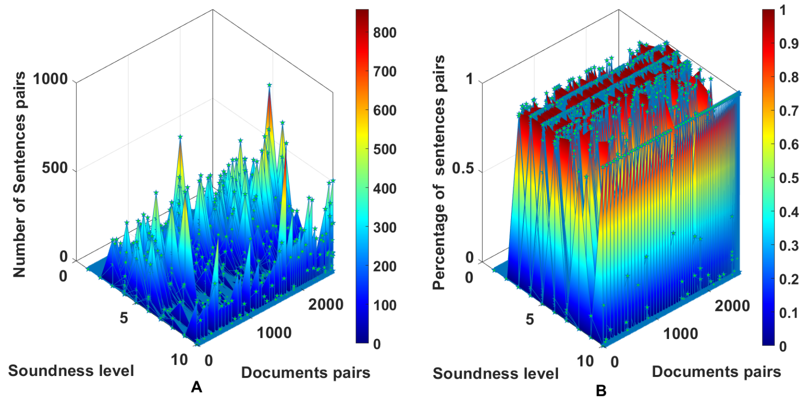

Figure 3: Distributions of dataset of 2,627 pairs considering pairs of sentence on and above the soundness level (noise with $\nu = 0.1$ is removed), using model all-MiniLM-L6-v2; (a) shows the distribution of similarity of pairs of sentences, (b) shows the distribution of the soundness level as defined in (5).

2 Criteria for tuning the fuzzy sets parameters and removing biases at the gold standard

A process for the creation of the dataset and gold standard in Subsection 5.1 implements the association of real values to the four labels described by the method in equations (6a) -(6d). Knowing that each model architecture is different and the training datasets are varied, distributions of similarity are expected to also vary with each model. These differences affect the level of noise ν in equation (2) for each model and the values associated with the method labels after the estimation of parameters of (6a) -(6d) which are produced by the process for the creation of the gold standard in Subsection 5.1. The following criteria are considered for tuning the parameters α, b, β, c, γ, and δ from (6a) -(6d):

1. The noise level ν may vary from the first decile to an upper decile within the range *[0,1]*, where ν accumulates the majority of low similarity of text pairs.

2. At the beginning, the parameters α, β, γ, and δ are distributed monotonically within the interval *[ν,1]*.

3. If the noise level ν covers a small number of text pairs with low similarity, then the ν may be updated to a greater value in the range *[0,1]*.

4. The parameter α for non-related pairs is affected directly by the increase or decrease of the noise level ν and should be updated according to the latter.

5. If a pair is labeled and gets the exact label at the assessment with the method, then no update should be applied since the assessment is accurate with the gold standard labeling.

6. If a pair labeled as non-related gets an assessment of concept-related when tested with the method, either the pair produces an overestimation or contains more similarity than expected. If the pair contains a concept-related similarity, then the pair is relabeled accordingly. Otherwise, the parameters α (and possibly β) is (are) out of context and should be updated with higher values in the range *[0,1]*.

7. If a pair labeled as non-related gets an assessment of same-topic when tested with the method, either the pair produces an overestimation or contains more similarity than expected. If the pair contains a same-topic similarity, then the pair is relabeled accordingly. Otherwise, the parameters α, β, and γ are out of context and should be updated with higher values in the range *[0,1]*.

8. If a pair labeled as non-related gets an assessment of identical when tested with the method, either the pair produces a full overestimation or contains more similarity than expected. If the pair contains an identical similarity, then the pair is relabeled accordingly. Otherwise, the parameters α, β, γ, and δ are out of context and should be updated with higher values in the range *[0,1]*.

9. If a pair labeled as concept-related gets an assessment of non-related when tested with the method, either the pair produces an overestimation or contains more similarity than expected. If the pair contains a non-related similarity, then the pair is relabeled accordingly. Otherwise, the parameter β (and α if too close) should be lowered since β is higher than expected.

10. If a pair labeled as concept-related gets an assessment of same topic when tested with the method, either the pair produces an overestimation or contains more similarity than expected. If the pair contains a same topic similarity, then the pair is relabeled accordingly. Otherwise, the parameter β should be increased; if the parameter γ is too close to β, then γ should also be increased. Additionally, the parameter c may be adjusted to reduce the fuzziness of the assessment.

11. If a pair labeled as concept-related gets an assessment of identical when tested with the method, either the pair produces an overestimation or contains more similarity than expected. If the pair contains an identical similarity, then the pair is relabeled accordingly; otherwise, the parameters β, γ, and δ should be increased monotonically.

12. If a pair labeled as same topic gets an assessment of non-related when tested with the method, either the pair produces an underestimation or contains less similarity than expected. If the pair contains a non-related similarity, then the pair is relabeled accordingly; otherwise, the noise is less than expected, and the parameter α must be decreased. Additionally, the parameters β, γ, and δ may be increased monotonically if exists room within the interval.

13. If a pair labeled as same topic gets an assessment of concept-related when tested with the method, either the pair produces an underestimation or contains less similarity than expected. If the pair contains a concept-related similarity, then the pair is relabeled accordingly; otherwise, the values of parameters c and/or γ are lowered. Additionally, if the difference between γ and β is too close to 0, then β is also lowered.

14. If a pair labeled as same topic gets an assessment of identical when tested with the method, either the pair produces an overestimation or the pair contains more similarity than expected. If the pair contains the identical similarity, then the pair is relabeled accordingly; otherwise, the parameter δ is updated with a greater value. Additionally, if the difference between γ and δ is too close to 0, then γ is lowered.

15. If a pair labeled as identical gets an assessment of same-topic when tested with the method, either the pair produces an underestimation or contains less similarity than expected. If the pair contains similarity as same topic, then the pair is relabeled accordingly. If the pair contains identical similarity, then the parameter δ should be lowered if near to 1; additionally, the parameter c may be updated to make the bell slimmer to reduce the bias, and or the γ should be lowered if the difference between γ and δ is practically zero.

16. If a pair labeled as identical gets an assessment of concept related when tested with the method, either the pair produces an underestimation or contains less similarity than expected. If the pair contains less similarity than identical, then the gold standard is changed accordingly. Otherwise, if there is a complete similarity, then the value of parameter δ is adjusted below the current value; the parameter γ is adjusted if the new value of δ is below γ; this last adjustment is also applied to β if the new value of γ surpasses the value of β.

17. If a pair labeled as identical and gets an assessment of non-related when tested with the method, either the result is a full underestimation or the pair contains no similarity (a complete false positive). If no similarity in the pair, then the gold standard is changed to non-related. If there is a complete similarity, then the value of parameter α should be decreased substantially, and the rest of the parameters β, γ, and δ should be decreased to distribute them in the range *[0,1]*, leaving a room between the parameter δ and the value 1.

18. Stop conditions: the application of the criteria described above will stop when one of two conditions are met:
    - Most pairs produce criterion 5
    - the parameters α, b, β, c, γ, and δ are focused in a small area of the range with no improvement after a series of iterations.

These criteria are integrated in a pseudocode described in Appendix 6. These criteria are applied to improve the accuracy of the models through a series of iterations.

3 Criteria for dataset compilation

The following criteria were used for the creation of the dataset:

1. Use the publicly available Wikipedia and other sources to retrieve long text documents.

2. The documents should be random in size in words; most of them should be of size at least 1,000 words, but the maximum number of words is not fixed.

3. Topics should be random. However, there are groups of documents with one degree of similarity: the documents of these groups should be concept-related or related to the same topic. There is no minimum or maximum number of groups of documents with similarities.

4. The topics are from different areas: science, history, politics, war conflicts, people, and other miscellaneous themes.

5. There are a few documents between 500 and 1,000 words to test the method's accuracy on medium-length documents.

The employed models for experimentation weren't injected with any context related to the dataset, and the gold-standard is a label for each pair of documents; the pairs of sentences are not labeled. The method should provide evidence of similarities between pairs of sentences and the label for the pair of documents according to the tuning of fuzzy parameters described in (6a)-(6d).

4 Dataset of Long Texts

This appendix describes the dataset used to analyze the similarity of random-size texts. The dataset consists of texts extracted from Wikipedia. The texts and their size in sentences and words are enlisted in Table 1 and Table 2 of this appendix. From the 72 documents in the dataset, 2,628 pairs, including testing a document against itself to create the similarity subset of identical documents (I), the rest of the types are obtained from the remaining pairs. This dataset can be retrieved from the repository

Table 1: Dataset of long-texts used for Analysis (Part 1).

Nr	Text	Sentences	Words
1	2022 Russian invasion of Ukraine.txt	512	12,272
2	2022 Russian invasion of Ukraine Brittanica.txt	309	6,564
3	Afghan Civil War (1992–1996) .txt	385	7,196
4	Afghanistan conflict (1978–present) .txt	433	9,165
5	Agricultural engineering .txt	56	716
6	Alexander the Great .txt	619	13,586
7	American Civil War .txt	887	16,827
8	Anatomy .txt	308	6,120
9	Antonio López de Santa Anna .txt	340	7,461
10	Aphasia .txt	278	5,919
11	Astronomy .txt	321	6,583
12	Astrophysics .txt	70	1,708
13	Assassination of John F. Kennedy .txt	354	6,877
14	Bible .txt	563	12,309
15	Biochemistry .txt	212	4,133
16	Biological anthropology .txt	31	648
17	Biology .txt	971	19,712
18	Chemical engineering .txt	68	1,234
19	Chemistry .txt	272	5,737
20	Christianity .txt	611	14,774
21	Cryptography .txt	347	7,447
22	Dentistry .txt	132	2,953
23	Deontology .txt	83	1,813
24	Don Quixote .txt	387	9,117
25	Earthquake .txt	266	5,533
26	Economics .txt	481	11,102
27	Emilio Mola .txt	57	1,099
28	Francisco Franco .txt	588	14,412
29	French Revolution .txt	660	15,962
30	Geology .txt	254	5,451
31	GuadalupeHidalgoTreaty_docsteach_org.txt	355	11,815
32	GuadalupeHidalgoTreaty_pbs_org.txt	52	935
33	Henry Ford .txt	449	8,543
34	History of slavery.txt	977	20,318
35	Interview Steve Allen on Vietnam.txt	552	6,189
36	Iran_Contra_BrownEdu.txt	766	8,158

Table 2: Dataset of long texts used for Analysis (Part 2).

Nr	Text	Sentences	Words
37	Iran–Contra affair .txt	349	8,076
38	Iraq War .txt	697	15,375
39	Islam .txt	434	9,360
40	James K. Polk .txt	681	14,422
41	Judaism .txt	515	11,698
42	Macroeconomics .txt	174	3,487
43	Mexican Revolution .txt	1,002	19,459
44	Mexican War of Independence .txt	347	7,870
45	Mexican–American War .txt	1076	18,231
46	Microeconomics .txt	190	3,818
47	New Spain .txt	672	16,986
48	Nikola Tesla .txt	489	10,861
49	Oliver North .txt	121	2,529
50	Parícutin .txt	124	2,457
51	Quantum mechanics .txt	806	7,168
52	Quran .txt	509	10,358
53	Ronald Reagan .txt	479	9,241
54	Russian Revolution .txt	410	9,018
55	Seismology .txt	98	2,058
56	Slavery .txt	771	16,493
57	Slavery in Africa .txt	472	10,964
58	Slavery in Asia .txt	236	4,936
59	Slavery in ancient Greece .txt	295	6,259
60	Slavery in ancient Rome .txt	270	5,834
61	Slavery in the United States .txt	1,097	23,603
62	Spain .txt	728	16,303
63	Spanish Civil War .txt	795	17,948
64	Spanish coup of July 1936 .txt	210	4,156
65	Spanish-American_US_LOC.txt	92	1,476
66	Spanish–American War .txt	633	12,106
67	Thomas Edison .txt	438	8,410
68	Torah .txt	223	5,646
69	Treaty of Guadalupe Hidalgo .txt	180	3,685
70	Volcano .txt	220	4,798
71	World War I .txt	1,076	24,004
72	World War II .txt	553	13,249

5 Criteria for the creation of the gold-standard

The gold standard of the dataset considers four labels: Identical, Same Topic, Concept-related, and non-related. Initially, these labels have no real value in the range *[0,1]*; instead, a human expert assigns the label to a pair of documents based on the common sense and experience of the expert. The contents of a pair of documents may differ from the initial label; for the sake of the accuracy of the gold standard, a set of criteria is established in the Subsection 2. The criteria is aimed to associate a value for each parameter of the fuzzy sets defined in (2), (6a)-(6d) and, at the same time, remove the labeling biases due to human common sense. The labeling can be produced without prior similarity estimation of pairs since the models are trained in advance, and the tuning of the assessment parameters.

5.1 Process of creation of the dataset and its gold standard

The creation of the dataset and its gold standard for testing the performance of the proposed method have the following steps:

1. **Selection of the long texts**. The dataset contains 72 texts ranging from hundreds of words to more than 24,000 words, with texts retrieved from Wikipedia. The topics of documents include wars, people, science, history, slavery, engineering, countries, religion, and events. From the set of 72 documents, 2,628 pairs are tested. [2]. For each pair, a label will be provided based on human commonsense. This label is the reference for testing the performance of the method using a model. The labels' values are described in the following step.

2. **Empirical selection of the values associated with the labels based on analysis distribution**. Based on the distribution of comparison of pairs of sentences throughout the two documents and the commonsense of the degree of similarity of the topics described by the pair of documents, a label is provided to the pair of documents (”IDENTICAL”, ”SAME TOPIC,” ”CONCEPT RELATED” or ”NON RELATED.”). A pair of documents is ”IDENTICAL” if the comparison at the level of sentences provided the maximum similarity. A pair is ”SAME TOPIC” if both documents describe a topic despite using different grammar structures and synonyms. A pair of documents is ”CONCEPT RELATED” if the documents describe different topics with common concepts. A pair of documents is ”NON RELATED” when their topics and concepts are completely different.

3. **Creation of the gold standard**. Consisting of a matrix of labels for the pairs from the documents. Based on common sense, each pair is labeled with one of the four labels described in the previous step. Since the detailed analysis of pairs is time-consuming, the gold standard is created without a systematic analysis of each pair of documents; instead, common sense is used. Generating the labeling by commonsense is described as follows:

   (a) Consider documents D1 and D2, and then a human expert decides whether they belong to the same topic. If the documents belong to completely different topics, then the pair's label is non-related; otherwise, proceed to the next step.

   (b) If the topics of both documents are related in meaning containing shared concepts; however, their contexts are different in space, time, or another dimension, then the label is assigned as concept-related; otherwise, proceed to next step.

   (c) If the topics are related in a close manner, describing a common context in time, space or semantics then the label is same topic; otherwise, proceed to the next step.

   (d) If both documents are deemed the same document in whole or part, then the pair is labeled identical.

   The previous process sets the initial label of each pair. However, the labeling may be established incorrectly due to scarce information in one or both documents. To remove biases, pairs of documents that are expected to have a high degree of similarity and experience a lower degree of similarity are tested in step 4, reviewed in their contents in the step 5 and updated in the step 6.

4. **Testing of the model performance**. The model's output is evaluated for a new selection of label values. At this step, an analysis of the differences reviews whether inaccuracies are due to biases on the label selection at the gold standard or model performance's biases imputed to the model's training.

5. **Review the falses, the underestimations and the overesimations**. In this phase, we review whether a bias exists in the gold standard or the assessment parameters should be updated.

[2]Note: It would be expected that almost 5,184 pairs are possible; however, testing document A vs. document B is the same result as testing document B vs. document A

6. **Updating of values or end of the process**. Using the testing output, either the labels of the gold standard (step 3) or the values of the assessment parameters are updated (step 2). The update consists of applying the central limit theorem on evaluations by pairs of sentences/chunks for each pair of documents to generate a new baseline.

6 Tuning of assessment parameters from gold standard

This section describes the process for tuning assessment parameters from a gold standard defined for document pairs while using a language model. Let a set of similarity labels set $L = \{L_1, L_2, .., L_n\}$, where $L_{i-1} < L_i < L_{i+1}$ for all labels except the lowest first L_1 and the greatest last L_n.The tuning process starts as follows:

1. The set of real parameters associated with each label $L_i \in L$ are initialized in a monotonically ascending set of values in the range [0,1].

2. Test each pair using the method with parameters and compare results against the Gold Standard. If the result is a match, there is no learning. If the comparison results in a sub-estimation $L_{gold-std} > L_{result}$, then the parameter associated with the gold standard label is lowered. If comparison produces an over-estimation $L_{gold-std} < L_{result}$, then the parameter associated with the gold standard label is increased. The matches, overestimations, and sub-estimations are recorded to statistically visualize the prediction performance regarding the gold standard. if the testing produces mainly matches at each variable, then the variables are already tuned; if an assessment of a variable produces mostly over-estimations, then the assessment variable should be increased; if a variable produces sub-estimations, then the variable should be decreased to achieve more match cases. If a variable produces all cases (overestimations, matches, and sub-estimations), then the accuracy is limited or biased by the training of the employed language model.

3. Retest the dataset with the new assessment parameters and compare it against the previous test. If the error is below a desired percentage or there is no improvement in learning, stop; otherwise, relearn as described in the previous step.

7 Complexity of algorithms

This section analyzes the complexity of Algorithm 1. The analysis is applied to each function in the algorithm. The implementation of the functions described in this analysis can be consulted at the repository at the link `https://github.com/omarzatarain/long-texts-similarity`. The repository content is described in the Subsection of code and availability.

SplitintoSentences: Let T1 be the number of words in the first document LT1 and LT2 in the second document LT2. The time to produce S the sentences from a document D with T words is linear O(T), since a sentence usually contains more than one word, therefore $\|S\| < \|T\|$. The total complexity of producing the sentences Sent1 and Sent2 from documents LT1 and LT2 is O($T1+T2$), where T1 and T2 are the number of words in LT1 and LT2.

GetEmbeddings: Producing the embeddings for each s $\in S$ requires $\|S\|$ iterations with a constant time C for the production of each embedding; therefore, the complexity is O($C \times S$) = O(S)

Block of comparisons: The systematic comparison of the two sets of sentences Sent1 and Sent2 and placing the results into the Matrix produces a complexity O($\|Sent1\| \times \|Sent2\|$).

GetSupport: Let a Deciles be an array of 10 elements; getting the support requires assessing up to the ten positions of deciles. Therefore, the complexity at the worst case is O(10).

GetSpanning: Let a Deciles be an array of 10 elements; getting the spanning requires assessing up to the ten positions of deciles. Therefore, the complexity at the worst case is O(10).

GetSoundness: Let a Deciles be an array of 10 elements; getting the soundness requires assessing up to the ten positions of deciles. Therefore, the complexity at the worst case is O(10).

ClassifyPair: Let the soundness the position of the decile with the greatest number of pairs with high similarity obtained through the function GetSoundness, the computation if the classification requires up to 15 computations for the four fuzzy sets I, ST, CR and NR, therefore, the complexity is O(1).

SelectRepresentativePairs: The selection of representative pairs retrieves the pair indices with the highest degrees of similarity by discarding the lower ones from a matrix of dimensions $\|Sent1\|$ and $\|Sent2\|$; the selection requires $O(\|Sent1\| \times \|Sent2\|)$.

SaveResults: This procedure requires recovering the analysis produced by the functions GetSupport, GetSpanning, and SelectRepresentativePairs and saving them to permanent storage. Therefore, the complexity is O(1).

Total complexity: Having in mind that $O(\|Sent1\| \times \|Sent2\|)$ and $O(T1 + T2)$, are the two major complexities, and $O(T1 + T2)$ is the complexity of preprocessing words into sentences, then the total, complexity is $O(\|Sent1\| \times \|Sent2\|)$ concerning to the generation of the embeddings of sentences (or chunks) and the comparison of the embeddings from both sets of sentences.

8 Tuning of parameters configurations

8.1 Configurations used on all language models

Table 3: List of Configurations for assessing the models performance.

	Assessment Classes					
	NR	CR		ST		I
Configuration	α	b	β	c	γ	δ
Baseline 0	0.3	0.2	0.5	0.2	0.7	0.9
Baseline 01	0.5	0.2	0.6	0.2	0.7	0.9
Baseline 02	0.6	0.2	0.64	0.2	0.7	0.9
MiniL12	0.55	0.2	0.65	0.2	0.75	0.9
MPNET 1	0.55	0.2	0.67	0.2	0.75	0.9
Glove 1	0.7	0.2	0.8	0.2	0.9	0.95
Glove 2	0.7	0.2	0.8	0.2	0.9	0.95
Longformer 1	0.9	0.2	0.94	0.2	0.95	0.98
Longformer 2	0.88	0.2	0.92	0.2	0.94	0.995
BART 1	0.86	0.2	0.88	0.2	0.90	0.98

Table 4: Assessment of configurations with model all-MiniLM-L12-v2

Configuration /		Confusion matrix				Performance			
Dataset	Class	I	ST	CR	NR	Prec.	Rec	F1	Acc.
Baseline 01 Benchmark	I	7	0	0	0	1.0	1.0	1.0	1.0
	ST	0	2	2	0	1.0	0.5	0.66	0.5
	CR	0	0	3	6	0.6	0.33	0.42	0.27
	NR	0	0	0	8	0.57	1.0	0.72	0.57
Baseline 02 Benchmark	I	7	0	0	0	1.0	1.0	1.0	1.0
	ST	0	1	3	0	0.0	0.0	0.0	0.0
	CR	0	0	3	14	0.0	0.0	0.0	0.0
	NR	0	0	0	0	0.0	0.0	0.0	0.0
Baseline 01 Dataset 72	I	71	2	0	0	0.98	0.97	0.97	0.97
	ST	1	27	111	35	0.93	0.15	0.26	0.15
	CR	0	0	77	425	0.39	0.15	0.22	0.12
	NR	0	0	5	1,873	0.8	0.99	0.88	0.80
Baseline 02	I	71	2	0	0	0.98	0.97	0.97	0.95
	ST	0	27	14	1	0.93	0.64	0.76	0.61
	CR	1	0	97	34	0.5	0.73	0.59	0.42
	NR	0	0	82	2,298	0.98	0.96	0.97	0.95

8.2 Tuning of parameters with model all-MiniLM-L12-v2

The baseline 01 is used for testing the method with the model all-MiniLM-L12-v2. Table 4 contains the confusion matrix and assessment of results; all classes contain pairs correctly detected, however, there are underestimations at classes CR and NR, therefore, criteria 18, 9, and 10 are applied to produce MiniL12_1 configuration. The assessment of MiniL12 1 configuration shows good performance on the classes I, ST, and NR, the CR class is detected in 50% of the cases, and the NR pairs are detected wrongly as the CR class. Since the model with this configuration has high uncertainty for setting the boundaries of CR and NR classes, criterion 18 of Subsection 2 is applied for the benchmark dataset.

The assessment of the dataset of 72 documents and 2,628 pairs with the model all-MiniLM-L12-v2 uses the Baseline 01; since there are overestimations at the classes ST, CR and CR, following the criteria 6, 10, and 14 are applied to define the MiniL12 2 configuration with higher values at parameters α, β, and γ. The assessment of the MiniL12 2 configuration with the model all-MiniLM-L12-v2; the performance improves with the configuration, especially for the CR and NR classes. However, several pairs indicate that the boundaries between CR and NR classes are fuzzy, therefore, criterion 18 of Subsection 2 is applied.

8.3 Tuning of parameters with model all-mpnet-base-v2

For the model all-mpnet-base-v2, Table 5 contains the configurations applied to the benchmark dataset and the dataset of 72 documents. The baseline 01 configuration produces good results when tested against the benchmark dataset and its gold standard. Since the classes I and ST are fully detected on the benchmark dataset. The MPNET1 configuration updates the parameters γ and δ by applying the criteria 6 and 10 from Subsection 2. The assessment using the MPNET 1 configuration on the benchmark dataset produces a better classification. However, the CR class has a greater underestimation than the previous configuration. Criterion 18 is applied since a good classification was achieved with the configuration and the dataset. The assessment results on the dataset of 72 documents and 2,628 pairs with the MPNET 1 configuration show a good balance of detected pairs in all classes; the class with the least performance was CR. Since a degree of fuzziness exists between classes NR-CR, CR-ST, and ST-I, criterion 18 of Subsection 2 is applied.

Table 5: Assessment of configurations with model all-mpnet-base-v2

Configuration/		Confusion matrix				Performance			
dataset	Class	I	ST	CR	NR	Prec.	Rec	F1	Acc.
	I	7	0	0	0	1.0	1.0	1.0	1.0
Baseline 01	ST	0	2	2	0	1.0	0.5	0.66	0.5
Benchmark	CR	0	0	3	6	0.6	0.3	0.42	0.27
	NR	0	0	0	8	0.57	1.0	0.72	0.57
	I	7	0	0	0	1.0	1.0	1.0	1.0
MPNET 1	ST	0	2	0	0	1.0	1.0	1.0	1.0
Benchmark	CR	0	0	2	0	0.4	1.0	0.57	0.4
	NR	0	0	3	14	1.0	0.82	0.9	0.82
	I	71	2	0	0	1.0	0.97	0.98	0.97
MPNET 1	ST	0	27	20	3	0.93	0.54	0.68	0.51
Dataset 72	CR	0	0	102	42	0.52	0.70	0.60	0.43
	NR	0	0	71	2,288	0.98	0.96	0.97	0.95

8.4 Tuning of parameters with model glove.6B.300d

The glove model is tested with the Baseline 01 configuration, the results are depicted in Table 6; the classes CR and NR are not detected, and the detections are concentrated in the I and ST classes. Therefore, by applying criteria 6, 7, and 10, the configuration Glove 1 is generated with higher parameter values. The results of Glove 1 show little improvement, however, the ST, CR, and NR still are not detected; therefore the Glove 2 configuration is defined by by applying the criteria 6, 10, and 14 of Subsection 2. The results of Glove 2 configuration do not improve the detection of the same topic class. Therefore, criterion 18 is applied. Model glove.6B.300d contains less sensitivity than model all-MiniLM-L6-v2, one possible cause is a less efficient training of glove.6B.300d. A comparison of the three configurations with the dataset of 72 documents and 2,628 pairs shows similar results to those achieved on the benchmark dataset. The results using the Baseline 01 configuration contain a concentrated distribution at the classes I and ST, and no pairs for classes CR and NR. The results using the configuration Glove show little improvement regarding the previous configuration and few pairs are correctly detected in the classes ST, CR, and NR. However, many pairs are wrongly assessed. The results using the configuration Glove 2 show worse performance than the previous configuration.

8.5 Tuning of parameters with model LongFormer

In the case of this large language model and the rest of LLMs, due to the time to produce the embeddings, the analysis was performed only on 998 pairs from the dataset of 72 documents, where the LLMs require memory and time resources of at least 2X for memory and 6X for time when compared to the sentence transformers. The parameter configurations are tested first on the benchmark dataset of 28 pairs, and based on observations, the dataset of 998 pairs is used from the original dataset of 2,628 pairs. Table 7 shows the performance of configurations Baseline 01, Longformer 1 and Longformer 2 on the benchmark dataset and the dataset of 72 documents with 998 pairs, none of the configurations exhibited sensitivity to differences.

8.6 Tuning of parameters with model BigBird

The BigBird model is tested with the LongFormer 1 configuration on the benchmark dataset, in Table 8, most pairs got an assessment of identical, although the δ parameter was set with a maximal value, which means that the model has no sensitivity to differences.

Table 6: Assessment of configurations with the model glove.6B.300d

Configuration/		Confusion matrix				Performance			
dataset	Class	I	ST	CR	NR	Prec.	Rec	F1	Acc.
	I	7	2	2	0	1.0	0.63	0.77	0.63
Baseline 01	ST	0	0	3	14	0.0	0.0	0.0	0.0
Benchmark	CR	0	0	0	0	0.0	0.0	0.0	0.0
	NR	0	0	0	0	0.0	0.0	0.0	0.0
	I	7	2	2	0	1.0	0.63	0.77	0.63
Glove 1	ST	0	0	3	8	0.0	0.0	0.0	0.0
Benchmark	CR	0	0	0	6	0.0	0.0	0.0	0.0
	NR	0	0	0	0	0.0	0.0	0.0	0.0
	I	7	2	2	0	1.0	0.63	0.77	0.63
Glove 2	ST	0	0	0	0	0.0	0.0	0.0	0.0
Benchmark	CR	0	0	3	8	0.6	0.27	0.37	0.23
	NR	0	0	0	6	0.42	1.0	0.6	0.42
	I	72	23	48	43	1.0	0.38	0.55	0.38
Baseline 01	ST	0	6	145	2,290	0.20	0.002	0.004	0.002
Dataset 72	CR	0	0	0	0	0.0	0.0	0.0	0.0
	NR	0	0	0	0	0.0	0.0	0.0	0.0
	I	72	23	48	43	1.0	0.38	0.55	0.38
Glove 1	ST	0	6	140	1,139	0.20	0.004	0.009	0.004
Dataset 72	CR	0	0	5	1,119	0.025	0.004	0.007	0.003
	NR	0	0	0	32	0.01	1.0	0.027	0.013
	I	72	23	48	43	1.0	0.38	0.55	0.38
Glove 2	ST	0	0	0	0	0.0	0.0	0.0	0.0
Dataset 72	CR	0	0	140	1,139	0.72	0.109	0.19	0.105
	NR	0	0	5	1,151	0.49	0.99	0.65	0.492

Table 8: Assessment of configurations for the benchmark dataset of 28 pairs with model BigBird and reviewed gold standard

Configuration/		Confusion matrix				Performance			
dataset	Class	I	ST	CR	NR	Prec.	Rec	F1	Acc.
	I	7	1	5	14				
Longformer 1/	ST	0	0	0	0	0.0	0.0	0.0	0.0
Benchmark	CR	0	0	0	0	0.0	0.0	0.0	0.0
	NR	0	1	0	0	0.0	0.0	0.0	0.0
	I	16	5	71	868	1.0	0.016	0.032	0.016
Longformer 1 /	ST	0	0	1	21	0.0	0.0	0.0	0.0
Dataset 72	CR	0	0	0	0	0.0	0.0	0.0	0.0
	NR	0	0	2	14	0.015	0.0875	0.030	0.015

8.7 Tuning of parameters with model BART

The BART model is tested with the Longformer 1 configuration with the benchmark dataset as shown in Table 9. The confusion matrix shows a highly unbalanced distribution, detecting all the pairs in class I or the NR class. Therefore, a new configuration, BART 1, is generated by applying criteria 9 and 12 from the criteria for tuning in Subsection 2. The BART 1 configuration applied to the model BART and the benchmark dataset produces overestimations for pairs of classes CR and NR. Based on behaviors from previous configurations on models Longformer and BigBird, the

Table 7: Assessment of configurations for the benchmark dataset of 28 pairs and the Dataset of 72 pairs with model Longformer

Configuration/		Confusion matrix				Performance			
dataset	Class	I	ST	CR	NR	Prec.	Rec	F1	Acc.
	I	7	0	0	0	1.0	1.0	1.0	1.0
Baseline 01	ST	0	2	4	14	1.0	0.1	0.18	0.1
Benchmark	CR	0	0	1	0	0.2	0.0	0.33	0.2
	NR	0	0	0	0	0.0	0.0	0.0	0.0
	I	7	0	0	0	1.0	1.0	1.0	1.0
Longformer 1	ST	0	0	0	0	0.0	0.0	0.0	0.0
Benchmark	CR	0	0	0	0	0.0	0.0	0.0	0.0
	NR	0	2	5	14	1.0	0.66	0.8	0.66
	I	7	0	0	0	1.0	1.0	1.0	1.0
Longformer 2	ST	0	0	0	0	0.0	0.0	0.0	0.0
Benchmark	CR	0	0	5	14	1.0	0.26	0.41	0.26
	NR	0	0	0	0	0.0	0.0	0.0	0.0
	I	16	0	3	46	1.0	0.24	0.39	0.24
Longformer 1	ST	0	0	0	0	0.0	0.0	0.0	0.0
Dataset 72	CR	0	0	0	0	0.0	0.0	0.0	0.0
	NR	0	5	7	857	0.94	0.91	0.87	
	I	16	0	3	46	1.0	0.24	0.39	0.24
Longformer 2	ST	0	0	0	0	0.0	0.0	0.0	0.0
Dataset 72	CR	0	0	63	723	0.85	0.08	0.14	0.07
	NR	0	0	8	134	0.14	0.94	0.25	0.14

model has low sensitivity to differences.

8.8 Tuning of parameters with model GPT2

The tuning of the model GPT2 starts with the Longformer configuration using the benchmark dataset. Table 10 shows the confusion matrix and performance of Longformer, the configuration, and the GPT2 model with the benchmark dataset. Although the parameter values are high, especially δ, most pairs are detected as class I, which means the model has low sensitivity to differences. In the case of 998 pairs from the 72 documents using the Longformer and the GPT2 model, scarce sensitivity is also encountered. Most pairs are detected as class I or ST, although the gold standard has a more balanced distribution.

9 Parallelization strategies for time performance

The computation core of the method is the comparison of the embeddings from sentences through the cosine similarity; this comparison is applied systematically on sentence pairs with complexity $O(S1 \times S2)$, the rest of the procedures are either the same complexity or linear. Therefore, this subsection analyzes parallel and serial versions of the cosine similarity. The embedding dimension sizes vary from 384 tokens to 4,096 tokens. The rest of the procedures in the algorithm In the case of sentence transformers, the dimension sizes are smaller than 768. In the case of LLMs, the greatest size is 4,096 (BART). Parallelized versions of the cosine similarity with CPU (using library numba), GPU (also library numba), and a serial version(using library numpy as used in the implementations of the method) are compared for pairs of embeddings with the sizes of the models were tested on the three versions as described in Table 11. Despite the algorithm can be parallelized, the dimensions of the embeddings are very small for getting advantages from specialized hardware resources.

Table 9: Assessment of configurations for the benchmark dataset of 28 pairs with model BART and reviewed gold standard

Configuration/		Confusion matrix				Performance			
dataset	Class	I	ST	CR	NR	Prec.	Rec	F1	Acc.
	I	7	0	0	0	1.0	1.0	1.0	1.0
Longformer 1/ Benchmark	ST	0	0	0	0	0.0	0.0	0.0	0.0
	CR	0	0	0	0	0.0	0.0	0.0	0.0
	NR	0	2	5	14	1.0	0.66	0.8	0.66
	I	7	0	0	0	1.0	1.0	1.0	1.0
BART 1/ Benchmark	ST	0	2	5	4	1.0	0.095	0.17	0.095
	CR	0	0	0	0	0.0	0.0	0.0	0.0
	NR	0	0	0	0	0.0	0.0	0.0	0.0
	I	16	4	59	548	1.0	0.025	0.04	0.025
BART 1 / Dataset 72	ST	0	1	13	335	0.2	0.002	0.005	0.002
	CR	0	0	0	0	0.0	0.0	0.0	0.0
	NR	0	0	2	20	0.022	0.90	0.043	0.02

Table 10: Assessment of configurations for the benchmark dataset of 28 pairs with model GPT2 and dataset of 998 pairs

Configuration/		Confusion matrix				Performance			
dataset	Class	I	ST	CR	NR	Prec.	Rec	F1	Acc.
	I	7	1	5	15	1.0	0.26	0.42	0.26
Longformer 1 / Benchmark	ST	0	0	0	0	0.0	0.0	0.0	0.0
	CR	0	0	0	0	0.0	0.0	0.0	0.0
	NR	0	1	0	1	0.071	0.5	0.125	0.066
	I	16	5	71	867	1.0	0.016	0.032	0.016
Longformer 1 / Dataset 72	ST	0	0	2	21	0.0	0.0	0.0	0.0
	CR	0	0	0	0	0.0	0.0	0.0	0.0
	NR	0	0	1	15	0.016	0.93	0.032	0.016

Table 11: Performance on parallel/serial cosine similarity on time (in seconds) with embeddings sizes used by the models.

	Model embeddings sizes				
Processing type	300	384	700	768	4096
Serial (numpy)	4.21×10^{-4}	8.21×10^{-4}	3.15×10^{-4}	2.88×10^{-4}	6.14×10^{-4}
GPU (numba)	2.09×10^{-1}	2.23×10^{-1}	2.4×10^{-1}	2.10×10^{-1}	2.27×10^{-1}
CPU (numba)	7.36×10^{-4}	9.53×10^{-4}	1.53×10^{-3}	1.69×10^{-3}	7.42×10^{-3}

10 Accuracy of large models through chunking

In this study, the proposed method is applied for testing the accuracy on Soundness (5) using different chunk sizes(instead of sentences) for assessing performance on sentence similarity for each large language model. The tested models are LongFormer, BigBird, GPT2, and BART (Unlimiformer). For demonstration, two tests are performed: one document against itself, and the document against a document speaking about the same topic. Comparisons of a document with itself at Table 12, the Longformer is the most inaccurate of the models as the chunk size increases, the rest perform well to

detect . The comparison of two documents considered as same topic in Table 13 shows a decreasing performance on pairs of documents related to the same topic and, with the smallest chunk size, all models overestimate the soundness of relationships, on the contrary, the analysis by splitting text into sentences produce a more conservative estimation. From results in Table 12 it observes that two models perform well on identical comparisons regardless of the size of sentences; however, in the case of the same topic in Table 13, all model experience a decline in the accuracy as the chunk size increases. There is an overestimation at the smallest chunk size of 16 words. This can be explained for several reasons: the first is that these models weren't necessarily trained with the pursued task in mind, the second is the lack of long-text datasets, and the third is the resources required to train models with longer texts. Therefore, the proposed method is unsuitable for working with fixed-sized chunks because chunking generates bigger biases than splitting texts by sentences, and additional strategies are needed to exploit the method and LLMs efficiently.

Table 12: Large models tested with the method on the similarity of a document against itself.

	Soundness using chunk sizes (tokens)							Split by
Model	16	32	64	128	256	512	1024.	sentences
Longformer	10	7	5	4	2	2	2	8
BigBird	10	10	10	10	10	10	10	10
GPT2	10	10	10	10	10	10	10	10
BART	10	10	10	10	10	10	10	10

Table 13: Large models tested with the method on the similarity of a document against another with same topic.

	Soundness using chunk sizes (tokens)							Split by
Model	16	32	64	128	256	512	1024.	sentences
Longformer	10	7	5	2	2	2	2	8
BigBird	10	8	7	6	5	5	5	9
GPT2	10	8	7	6	5	5	5	9
BART	10	8	7	6	5	5	5	9
